# Music and Mosquitos

**Published:** 1901-11-16

**Authors:** 


### MUSIC AND MOSQUITOS.

A good deal has lately been made of the fact that mosquitos are attracted by certain colours, but the much more interesting fact has recently been observed that they respond to certain sounds. Mr. Brannan, of the Public Works Department, Jamaica, says that by making a continuous whoop or hum he can gather swarms of them round his head, and now Sir Hiram Maxim goes a step further, and says that it is the male mosquitos that are so attracted. Some rather large electric lamps were erected at an hotel at Saratoga Springs to illuminate the grounds, and one of these lamps gave out a humming noise. The note was practically constant, and no adjustment of the carbons had the least effect upon it. This lamp always attracted the mosquitos, but only the males. When the lamps were started every male mosquito would at once turn towards this lamp, and then fly in the direction whence the sound proceeded. The female mosquitos, which are easily distinguished from the males, were far the more numerous, but it was only the males which seemed attracted by this musical lamp, and Sir Hiram Maxim suggests that as the pitch of the note was almost identical with that of the buzzing of the female mosquito the male took the sound to be that made by the female. He adds that if one obtains a tuning-fork which gives off a musical note as much like the hum of the female mosquito as possible, and strikes this tuning-fork within twenty feet of a male mosquito, he will at once turn about, face the music, and erect the two little feathers on his head, something after the manner of a cockatoo. He suggests that the two little feathers on the head of the male mosquito act as ears, and vibrate in unison with the music of the female or of the lamp as the case may be. It is an interesting observation.

